# A Qualitative Analysis of UK Wetland Visitor Centres as a Health Resource

**DOI:** 10.3390/ijerph18168629

**Published:** 2021-08-15

**Authors:** Jonathan P. Reeves, Conor H. D. John, Kevin A. Wood, Phoebe R. Maund

**Affiliations:** 1 Ecosystem Health & Social Dimensions Unit, Wildfowl & Wetlands Trust (WWT), Slimbridge, Glos GL2 7BT, UK; kevin.wood@wwt.org.uk (K.A.W.); phoebe.r.maund@gmail.com (P.R.M.); 2 School of Psychology, Cardiff University, 70 Park Pl, Cardiff CF10 3AT, UK; conorj1995@gmail.com or

**Keywords:** wildlife tourism, motivation, relaxation, biodiversity, spiritual wellbeing, attention restoration, green space, blue space, pro-environmental behaviours, connection to nature

## Abstract

The health benefits associated with spending time in natural environments have been highlighted during the COVID-19 pandemic. Lockdowns and restrictions to safeguard public health have exacerbated the pre-existing mental health crisis and rise of non-communicable diseases. Thus, the importance of nature as a health resource has been elevated, hastening calls for a better understanding of how health benefits might differ across user groups and nature provisions. In this regard, urban green spaces have become the greatest research focus; however, blue spaces, especially inland freshwater (e.g., wetlands), remain less studied. First-hand user experiences are also under-represented. This exploratory study examines the motivations and benefits of active wetland centre users in the UK, both during and after visits. Responses to three open-ended questions were collated online from 385 participants, and a qualitative content analysis was conducted based on an existing taxonomy from users of urban green spaces. The results showed strong motivations to visit due to the biodiversity at the site (mainly the birdlife), while less tangible nature (e.g., fresh air) and amenities were also important. In contrast to other studies on natural environments, physical activity was a less influential motivation. Salient derived effects included positive and intensely positive emotions, relaxation and mental restoration. After visits to wetland centres, feelings of vitality and satisfaction were the most prominent effects that emerged. For decision-makers looking to leverage inland blue spaces for public health benefit, our results highlight the broad range and relative prominence of the reasons for use and the associated perceived health benefits derived by users of UK wetland centres. They highlight how biodiversity, abiotic nature and good amenities are important qualities to consider when planning, managing and encouraging people to use natural environments for health benefit, qualities that may also provide important environmental co-benefits.

## 1. Introduction

The diverse health benefits derived from spending time in a natural environment are supported by a growing body of evidence [1,2,3]. Benefits accrue through improvements to psychological wellbeing [4,5,6], physical health [7] (e.g., through reductions in allergies and respiratory diseases [8], and protection against high/low blood pressure [9]) and opportunities for exercise [10,11,12]. As such, the question of how nature might be leveraged to tackle growing public health concerns regarding declining mental health [13] and other non-communicable diseases (NCDs, e.g., cardiovascular disease, cancers and diabetes) [14,15] is now a priority in both practice [16,17,18,19,20,21] and policy [22], as the economic costs associated with poor health escalate [23]. COVID-19 has only served to amplify this agenda. The pandemic has exacerbated existing public mental health and NCD prevalence [24,25,26,27,28]. However, it has also highlighted the services that natural spaces provide to human health, proving a powerful tool in mitigating the negative mental health consequences of physical distancing [29]. Outdoor spaces have also helped limit virus spread [30]. Thus, the pandemic—plus the growing application of nature-based health interventions [16,17,18,19,20,21]—has heightened the urgency to improve our understanding of how and why different types, qualities and experiences of natural environments influence wellbeing differently [1,3,5,21], especially in urban environments [31,32,33,34,35].

The way we use and manage nature has consequences for people’s health [31,36,37]. Traditionally, our management of nature has followed utilitarian (‘nature for people’, e.g., parks/urban green spaces) or protectionist principles (‘nature for itself’, e.g., nature reserves/protected areas) [38], but encouraging people to visit nature brings more wildlife disturbance [39], so trade-offs must be made between the benefits to people and biodiversity [40,41]. Urban green spaces have attracted the greatest research focus in relation to health, and the evidence reveals a broad range of health benefits [31,42,43,44,45,46]. For urban, free-to-use and open access spaces, health and wellbeing is a central benefit provided to people [47]. Their proximity/locality is key to facilitating healthful behaviours such as encouraging physical activity [43,48,49,50], socialising [51] and dog walking [52]. Conversely, protected areas are often characterised by restrictions to access and development primarily to prioritise biodiversity over other ecosystem services, e.g., recreation. While this has meant that they have received comparatively less attention, their potential as a health resource should not be overlooked. For example, the global yearly value of protected areas for individual human mental health and wellbeing was estimated at US $6 trillion, an order of magnitude greater than their tourism value [53]. Protected areas are often used by individuals with specialist interests in wildlife (e.g., bird watchers), and these experiences can foster feelings of awe, wonder and privilege induced by nature’s form, performance and biodiversity, along with deep nature connections [54]. Broad psychological/emotional, social, cultural and environmental benefits can accrue through experiences with nature in protected areas [55], from refuge from everyday life [56] to mental and spiritual health improvements [57]. Protected areas also bring broader benefit by conserving societal connection to nature and by increasing pro-environmental behaviours [58].

Blue spaces are another important yet understudied (in comparison with green spaces) environment type. Positive associations of the proximity of blue spaces for health have been reported [54,58,59,60]; however, existing research has tended to focus on coastal blue spaces, inducing calls for more research on inland, freshwater blue spaces (e.g., wetlands, rivers, lakes or canals) [59,60], although this imbalance is beginning to be redressed [12,61,62]. Possible non-material mechanisms to wellbeing through blue spaces include mitigation (from environmental harm), instoration (building capacity) and restoration (capacity restoration) [63]. For instoration, encouraging increased physical activity (relative to green spaces) may be an important mechanism [12,64,65,66], as is the opportunity to build positive relationships [67,68] and nature connectedness [69]. Restoration may be achieved either by reducing stress [70] or by implementing cognitive restoration [71,72]. Wetlands specifically may encourage stress recovery (from just 10 min exposures), particularly for individuals who experience high levels of self-reported stress [73]. Restoration through blue spaces is derived through high levels of preference, perceived restorativeness and positive affect relative to green spaces [60,74,75,76]. Research investigating visits to marine and coastal environments found that higher perceived biodiversity leads to people feeling more restored and happier during their visits [77]. The importance of restoration and affect has also been demonstrated for urban wetlands [78] and through a meta-analysis of blue space research [79]. The ability of wetlands to provide restoration is especially important where blue spaces are to be operationalised for health interventions [20]. Wetlands too are especially beneficial to health with regard to environmental mitigations (e.g., threats from climate change, flooding and other natural disasters [80,81,82]), so they may be a key environment in delivering ‘win–wins’ and multiple benefits to society [83].

In the UK, there have been notable increases in visits to natural environments for health reasons in the last decade [84], moving it up on the political agenda [85,86]. As such, there is a growing policy need for a better understanding of the complex ways in which people engage and become disengaged with natural environments [87,88]. Capitalising on this growing public interest, and finding middle ground where natural environments deliver for both human health and biodiversity, is a key aim for conservation [3,83]. One mechanism that would facilitate this would be for more biodiverse environments to be proven as being disproportionately better for human health [83]. However, despite positive associations (especially in relation to human–bird contact and bird species richness [5,53,54,55,56,57,58]), studies often return nonsignificant findings and are limited in terms of causation [3,6], again, emphasising the need to better understand the contributions of different nature provisions to health.

In this study, we focus on wetlands and health. We present evidence from UK wetland visitor centres. These are protected areas (with conservation designations) but with extensive visitor infrastructure (see Section 2.2), so within the suite of UK nature provision, they may be conceptualised as sitting between free-to-use urban parks and protected national nature reserves, thus providing a fresh perspective on how varying nature provision contributes to health. Many studies focus on proximity [64,89,90] using panel data [12,90,91] and focus on green space [5,50,92] and urban usage [32,89,93,94]. Here, we respond to calls to focus on users’ own articulation of their nature experiences [85,94,95,96] by conducting a qualitative (content) analysis of wetland centre users’ own motivations and derived effects (collected via online survey) during and after visits. We adapt a framework from UK urban park spaces [43] to develop a taxonomy specific to wetland centre users to help understand wetland centres’ contributions to health. We draw comparisons to users of urban green spaces and discuss findings in the context of prevalent nature–health theories. We find evidence that wetland centre users are strongly driven by the biodiversity, abiotic nature and amenity value of the space, leading to broad benefit in a range of wellbeing domains. We also find evidence that the wetland centres meet restorative environment criteria, as defined by attention restoration theory [71,72].

## 2. Materials and Methods

### 2.1. Data Collection and Procedures

Three open-ended questions (adapted from Irvine et al. [43]) were self-administered in an ex situ online questionnaire to capture wetland centre users’ descriptions of motivations for use of the wetland centres: “What are your main reasons for visiting a WWT Wetland Centre?” (hereafter referred to as ‘motivations’). Derived effects during visits were explored with “Thinking about when you are at a WWT Wetland Centre, please describe how you feel during your visit” (hereafter referred to as ‘during-visit effects’), while derived effects after visits were explored by “Thinking about when you leave a WWT Wetland Centre, please describe how you feel after your visit” (hereafter the ‘post-visit effects’).

These questions were asked first, followed by a series of closed-ended questions capturing participant demographics, site use and information regarding affiliation to the organisation. The research materials were piloted with nine participants and resulted in minor changes to two multiple choice questions. All participants gave informed consent for inclusion in the study. The study was conducted in accordance with the Declaration of Helsinki, and the protocol was approved by the Human Research Ethics Committee of the Wildfowl and Wetlands Trust (WWT0325112019). The data were collected over two weeks during March 2020.

### 2.2. The Wetland Centres

**Figure 1:**
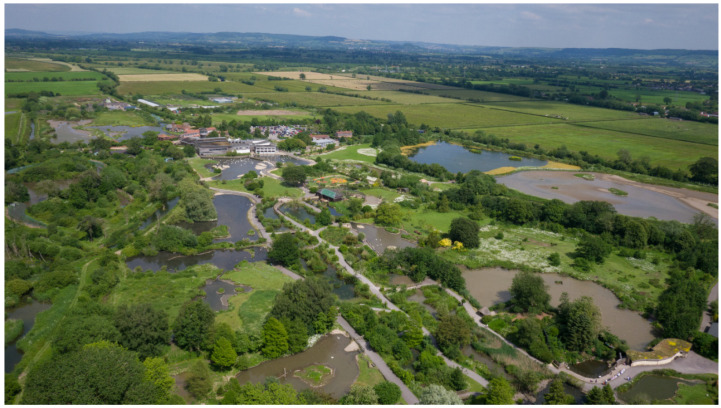
Slimbridge Wetland Centre, Gloucestershire, UK; an example of a wetland centre used in this study. Wetland centres function as both visitor attractions and protected natural areas. The central grounds area (47 ha at Slimbridge) features a visitor centre—including a cafe, an education centre and offices. The adjacent grounds feature a captive wildfowl collection, bird hides and children play areas. The protected natural areas (270 ha at Slimbridge; Site of Special Scientific Interest (SSSI), a Special Protection Area (SPA) and a Ramsar Site) surround the central visitor centre area and grounds. Image: WWT.

Wetland centres are characterised as paid-for visitor centre attractions with a wide variety of amenities in the central areas of the sites (e.g., cafes, education centres, classrooms, meeting rooms and offices, children’s play areas; Figure 1). Seven out of ten of the sites contain a captive wildfowl collection. Surrounding the centres are protected reserves with at least one or more environmental protection designations (e.g., SSSI, Ramsar site and/or SPA), with hides for wildlife watching.

### 2.3. Participants and Survey Responses

The survey was sent to 1000 randomly sampled members of a panel of 1662 wetland centre visitors. The panel consisted of 31.8% non-members, 58.1% members and 10.2% life members. Membership allows for unlimited access to all wetland centre sites, while non-members pay per visit. A 50% response rate was achieved (*n* = 496), and 111 incomplete, non-consenting or erroneous responses were removed, leaving 385 complete responses for analysis. The majority of the sample were White British or Irish (97%), and over 60 years of age (62%), 51% were female, 49% male (Supplementary Materials Table S1). WWT members made up 91% of respondents. Over two thirds (73%) of respondents had visited a wetland centre within the last three months, with 42% visiting within the last month.

### 2.4. The Coding Process and Analysis

An iterative content analysis process was conducted by two authors (J.R. and C.J.) [43,97,98,99,100]. The 385 participants’ full text responses were separated into short phrase meaning units (MUs) [97], e.g., “I’d say I’m mostly happy and relaxed and glad for a nice time having been had” [P157] and produced three MUs: “mostly happy”, “relaxed” and “glad for a nice time” (Table S2). Where participants offered more than one MU within the same code (i.e., a double code), only one MU was included. This allowed for the quantification of MUs to inform on the relative prominence of motivations or effects across codes, themes and domains [43]. The results (in Tables) are presented as total MUs for all responses per question. The MUs were iteratively checked by both coders to discuss the queries and ambiguities and to check the accuracy and credibility [97]. MUs that contained motivations but appeared in answers for during-visit effects and post-visit effects were moved to the motivations data set and vice versa.

The separated, checked and finalised MUs were coded based on the taxonomy (domains, themes, codes and sub-codes) and method of Irvine et al. [43]. Additional codes/themes/domains were created for MUs that did not fit this existing taxonomy. Both researchers jointly coded the first 20% of MUs, and the interrater reliability was calculated to ensure consistency between coding. Good agreement (range 0.706–0.86) for each question was demonstrated using Cohen’s kappa value [101] (Table S3).

Following satisfaction with a good interrater reliability, each coder separately coded 40% of the remaining MUs. Both coders iteratively reviewed and discussed the taxonomy structure, combining synonyms and redundant codes to achieve a final taxonomy (in Tables). A small number (approx. 1%) of erroneous or irrelevant MUs were removed from the analysis. For derived effects, there were ambiguities relating to pleasure and enjoyment; therefore, distinctions were made based on the object of the pleasure. For example, expressions of pleasure towards the site, e.g., “[…] pleased that the London WWT site is in Barnes”, were coded as place attachment, whereas pleasure from the experiences, e.g., “enjoy using all my senses”, were coded as affective.

## 3. Results and Discussion

The present study strengthens the wealth of existing evidence demonstrating that exposure to natural environments can lead to range of health and wellbeing benefits [2], especially in terms of mental health [4]. The survey responses were clustered into seven motivational domains, seven during-visit effects domains and eight post-visit domains. The significance of these domains are discussed below with exemplar quotes from responses provided in quotation marks with the participant number in square brackets e.g., [P123]. To inform on within-question prominence, the number of MUs assigned to each code/theme/domain are presented along with a percentage of the total MUs for the overall question.

### 3.1. Motivations for Use

A total of 1522 MUs were identified from responses to the question “What are your main reasons for visiting a WWT Wetland Centre?” and grouped into the following seven domains: space qualities, cognitive, physical, social, children, spiritual and values (Table 1).

#### 3.1.1. Space Quality Motivations: Biological and Abiotic, Intangible Nature

The most cited domain was space qualities (946 MUs, 62.2%). The wetland centres are sites of conservation importance and, therefore, have a focus on providing ecologically rich experiences with nature. This proved to be the most prominent motivating factor. For example, bird and other wildlife watching were the most cited responses, echoing recent studies that have highlighted the importance of bird species richness to life satisfaction (to a similar degree to income levels) across Europe [102]. Those that birdwatch often added their interest in particular bird groups, e.g., “wildfowl” [P347], “the swans” [P221] or “real geese” [P21], as an additional motivator. Bird migratory (seasonal) behaviour was often mentioned (35 MUs, 2.3%), e.g., “[…] to see the migratory wildfowl from the Arctic in the winter” [P51]. Only two MUs were related to species rarity (a previously cited motivation for wildlife viewing [103,104]); overall, the theme was less about specific birdwatching goals but rather “to see interesting birds in their natural environment possibly ones we cannot readily see locally” [P3] and to “be surprised by something new” [P238], “unexpected” [P158] or “unusual” [P132].

The motivation to see more general wildlife was a common motivation (129 MUs, 8.5%), expressing a more general wish to “see different wildlife” [P135] or “to enjoy nature” [P202]. Specific birding goals were often augmented with secondary non-avian wildlife motivations, e.g., “apart from birds which I am particularly interested there is so much more to observe” [P63].

Less tangible, abiotic features of nature, such being outside (58 MUs, 3.8%), the fresh air (45 MUs, 3.0%) and seasonal changes in nature (32 MUs, 2.3%), were important factors, often cited as secondary/tertiary reasons for visiting, alongside wildlife viewing, e.g., “I love birdwatching and getting outside” [P73]. Being outside and fresh air were often mentioned in tandem, reflecting a desire for escapism and refuge, e.g., “To get some fresh air and get away from all the ‘screens’ in the house” [P79]. In a similar tone to the mentions of bird migratory patterns, the seasons code recorded an awareness and desire to “experience the rhythm of the seasons” [P170], supporting other work on the importance of cyclical natural processes in bringing people to wetlands [105]. The desire for participants to be in and to view pleasant environments (24 MUs, 1.6%) and to be in and near wetlands/water (12 MUs, 0.8%) was also captured. The protected nature code (9 MUs, 0.6%) reflected a sense of appreciation from participants that a “true” [P137], “unspoilt” [P33], “natural” [P306] yet “managed” [P307] setting was being preserved for participants.

Our results suggest that the nature richness is a key driver of wetland centre use, aligning with natural spaces more broadly. A study on the motivations associated with the use of New York City’s ‘natural’ (i.e., forests, meadows and wetlands) versus landscaped (i.e., playgrounds, lawns and playing fields) parkland areas also reflects this trend [48]. Users of natural areas cited nature-related reasons as being the second-most important driver for use (22% of people), whereas for landscaped parks, nature ranked seventh (9.8% of people) [48]. By contrast, for public access UK parks, the potential for physical pursuits is a leading motivation [43,106]. For parks, while nature remains a prominent motivator, it is the intangible, abiotic aspects—such as being outside and in fresh air—that are well cited; biological nature is a notable low-level motivator [43]. While biodiversity provision varies between protected areas and public urban green spaces, the abiotic, intangible aspects of nature seem to have universal importance in driving use, the awareness of which has been heightened throughout the COVID-19 pandemic [29,30]. This importance is also now reflected in the increased recognition of abiotic nature within ecosystem service frameworks [107]. Our finding that the nature richness of the wetland centres is a key driver of use also relates to emerging evidence correlating species richness and habitat diversity to restoration and wellbeing benefits [5,44,108,109,110]. Frequency of use is also a factor. Around half the survey respondents visited wetland centres less than five times per year, and this aligns with evidence from urban green spaces, in which infrequent users are more likely to state motivations associated with the quality of the space [49].

Further non-biological motivators involved the sites’ features, and this theme reflected the wetland centres’ function as a visitor attraction. This was the second-most prominent motivational theme (353 MUs, 23.2%). Visitor amenities and aspects of site management designed to attract visitation were commonly expressed as secondary or tertiary responses to a nature-related primary motivator, e.g., “I get a massive thrill out of seeing wild birds [...] Wetland Centres offer good hides, easy access and informed company of local guides and visitors [...]” [P96]. Peace and quiet were also motivators (52 MUs, 3.4%), as was proximity, safety, the absence of nuisances (e.g., dogs/bicycles) and accessibility of the sites (good walkways and provision for the less mobile). The combination of motivations regarding high-quality biological nature and good facilities is supportive of the findings from Hong Kong in which regular use of urban blue spaces was more likely for users who thought that the space had good wildlife and facilities [62]. Similarly, users of natural wetland areas in New York also ranked amenities relatively highly as a motivation to visit [48].

#### 3.1.2. Cognitive Motivations

Although dwarfed by space qualities, cognitive motivations was the second-most prevalent motivation domain (171 MUs, 11.2%). This included people a) seeking opportunities for mental pursuits (100 MUs, 6.6%), e.g., photography or learning about wildlife/conservation, and b) seeking mental and attentional restoration [71,72], i.e., to be away from cognitively depleting environments or activities (46 MUs. 3.0%). Here, respondents described escaping the stresses of modern working life, e.g., “(to) disconnect from usual pace of life” [P49]/“seek some solace from a busy world” [P252]. The mental health code (25 MUs, 1.6%) collated general mentions of benefits for mental restoration and wellbeing, e.g., “mental clarity” [P69] or “mental exercise” [P80], as well as specific mentions of self-management for existing mental health conditions, e.g., “I have problems with anxiety and depression. My wellbeing is greatly improved when I visit a WWT Wetland—particularly during the winter” [P133].

A need for refuge has been reported for users of natural urban forests and wetlands in the New York city area [48]. By contrast, for park spaces (through open interview), escaping cognitive stress did not emerge as a motivator in UK parks [43]; however when presented as a check-box survey [111,112], through focus groups [113] or online survey [114], these themes emerged. Although an important motivator for more natural spaces, the picture is more unclear for urban green spaces and is variable across methodologies. The motivations regarding ‘being away’ may also be inclusive of participants’ desires for privacy [115], suggesting that restoration may be mediated by the enclosure afforded by wilder spaces, i.e., with bushes and trees [116,117]; however, with their open landscapes, this seems unlikely to hold for wetlands.

#### 3.1.3. Physical Motivations

Physical motivations were the third most commonly cited motivational domain (161 MUs, 10.6%) and include themes of physical restoration (97 MUs, 6.4%) and physical pursuits (64 MUs, 4.2%). The majority of restoration intentions were to “have a relaxing time” [P183], while sixteen respondents recognised and mentioned “the great wellbeing benefits” [P80]. To increase physical activity is one hypothesised mechanism by which natural environments bring about health benefit [1,118]. Physical pursuits were a lower-level motivator in our data and a less prominent driver of visits to wetland visitor centres for potential health benefits. This highlights the trade-offs that exist for nature managers looking to deliver multiple ecosystem services. The protections in place that prevent free roaming and wildlife disturbance secure biodiversity gains and, as noted above, attract nature-orientated visitation. However, the same protections are also a barrier for physical activity and its associated health benefits [43]. Indeed, where there is less enforcement of roaming restrictions in protected wetlands, physical pursuits such as walking and cycling are a prominent motivator for recreational use [119]. Dog walking is also restricted at the wetland centres, and this is a well-established method of physical activity in natural environments [52]. By contrast, for open, unrestricted urban parks, physical pursuits are frequently primary motivators for use [43,48,49,50]. This finding is also likely related to the strength of the nature-orientated motivations within our data. Nature-motivated users of freshwater blue spaces have been shown to favour psychological benefits (relaxing/destressing) over physical exercise benefits, while those not nature-orientated prefer physical benefits over psychological [61]. These findings emphasise not only the importance of understanding the drivers and motivations of users to secure health benefits from green and blue space access but also the importance of balancing access for health reasons with the protections required to promote wildlife.

#### 3.1.4. Social Motivations

The fourth motivation domain was social motivations (71 MUs, 4.4%), with the majority of these references relating to “family time” [P190], “time with friends” [P50] or visiting with a partner. The friendliness of other visitors was noteworthy, often in regard to the hides, as was the equally referenced motivation “to meet like-minded people” [P137]. These social interactions are another method by which health benefits accrue. Nature is thought to be a suitable arena for social interaction [1], and the need for social interaction can be especially important for older users of green spaces [120]. Sonti et al. [48] found similar levels of endorsement (3.9% of people asked) for socially focused motivations to use natural urban wetlands in New York. UK urban park use also saw around 4% endorsement (18/445 motivational statements) regarding the social domain [43]. An openness and good level of maintenance of nature spaces may be an important aspect in leveraging social interactions. In urban neighbourhood park spaces, open greenness is important for socialising [11], whereas in US state parks, well-maintained and developed outdoor areas are preferred to provide better social support than dense vegetation [121]. This may be related to an ability to accommodate larger groups of people compared with wilder park spaces [121]. As above, our results also demonstrated the importance of site features as a motivation to visit (e.g., bird hides and cafes), suggesting an increased prospect for social interactions that come with these amenities. This is a factor to consider where nature managers aim to increase engagement with underrepresented demographics or audiences to whom social interaction is especially important.

#### 3.1.5. Children Motivations

For the children domain (67 MUs, 4.5%), some motivations were simply stated as “to bring the children [P151]”, but equally prominent was a desire for the children to “commune with nature” [P154]. The child development theme (21 MUs, 1.3%) captured parental drives to encourage an “interest in the natural world and environmental issues” [P284]. This has political relevance as questions surrounding how we can improve connections with nature in children moves up on the political agenda [22]. This discussion relates not only to potential pro-environmental behavioural gains [122,123] but also to child development [124,125].

#### 3.1.6. Values and Spiritual Motivations

New domains were added (values and spiritual) to the parks taxonomy [43] to capture motives to support conservation and to connect with nature. These two domains were evenly endorsed (56 MUs, 3.7% and 53 MUs, 3.5%). For the majority, connections with nature were expressed literally as “to connect with nature” [P14,29,54,183], with deviations including, e.g., “being close to” [P12,37,83,136,171,217,232,234,244,312,320], “amongst” [P158,99,134] or “engaged with” [P33,107,112,382] nature. Other expressions of nature connectedness included “(I’m) searching for bigger picture nature” [P129] and “for me it’s a spiritual experience” [P213]. Support for conservation was interpreted as an altruistic and humanitarian value (a commonly cited motivator in conservation volunteering [126,127]) and was demonstrated to be a desire to provide “support for the organisation” [P353] or “to give back” [P214]. “Loyalty” [P129] for the organisation was expressed as an appreciation of “what the Trust is doing for conservation” [P56]. 

By contrast, for UK park use, neither spirituality nor values were recorded as motivators [43]. Although clear differences in environmental provision between studies must be taken into account, our findings of spiritual and value-driven motivations may be reflective of a shift in the public narrative over the last decade regarding the need and benefits of connecting with nature for wellbeing and societal gains [128,129]. Furthermore, the presence of these motivations for visitors to wetland centres supports existing evidence that a greater connection to nature is facilitated by environmental quality [130]. Additionally, more regular nature experiences [131,132]—as well as living in and near protected areas [58]—fosters increased pro-environmental behaviours [133].

Although the current work is limited in causally linking nature connection and conservation support, positive relationships between recreational nature visits/appreciation and pro-environmental behaviours [134,135] and across a range of socio-demographics [136] have been demonstrated. Our results demonstrate that the spirituality domain (and connection with nature) is both a motivator and derived effect; however, the values domain (supporting conservation) was only (significantly) recorded as a motivation, suggesting that, for some participants, it may be less important to derive reciprocal benefits from the financial support provided by their visit or membership, a finding of relevance to the emerging field of conservation marketing [137,138].

### 3.2. Derived Effects—During and after Visits

Two questions were asked about how participants feel during and after visits to wetland centres. The 1048 during-visit derived-effect MUs were clustered into the following domains: affective, physical, cognitive, spiritual, place attachment, social and global wellbeing (Table 2). For post-visits, 843 derived-effect MUs were clustered into the same seven domains with the addition of the values domain (Table 3). The salient differences between during- and post-visit effects are discussed below.

#### 3.2.1. Affective Derived Effects

The affective domain was the most referenced (292 MUs, 27.9%), by comparison, for UK park users, and this domain was the second-most referenced (behind the physical domain) [43]. This domain relates to recalled emotions within respondents themselves induced by the experience of the visit. Positive emotions was the most prominent theme across all responses (192, 18.3%), and feeling “happy” [e.g., P215] was the most frequent within-theme response. Pleasure and enjoyment for a range of site- and non-site-related reasons were stated, e.g., enjoyment of nature, wildfowl, the different seasons or enjoyment of others’ enjoyment. Other positive emotions included anticipation (27 MUs, 2.6%) mainly for potential wildlife sightings. Intensely positive emotions (93 MUs, 8.9%) were expressed to a level half that of positive emotions. These were expressed through excitement (65 MUs, 6.2%) at the wildlife to be seen; great pleasure (17 MUs, 1.6%), which included feeling great, delight, wonderful, elated and joy; and amazement at nature (11 MUs, 1.0%), e.g., “Amazement at the beauty and diversity of nature” [P257]. These results mirror other studies on the motivations and effects of visits to natural environments, where positive and intensely positive remarks were the most prominent derived effects [43,139]. This finding on the prominence of positive affective and physical states of feeling happy, pleasant and relaxed is supported by meta-analyses on natural environments and affect [140]. More specifically for wetlands, wellbeing has been shown to be promoted through positive affective responses in residents and visitors to nearby urban wetlands [78].

The affective domain was less prominent as a post-visit effect (163 MUs, 19.2%; Table 3). Feelings of anticipation, and positive and intensely positive emotions (e.g., excitement and amazement) decreased. Responses regarding anticipation shifted to a focus on processing the days’ activities, e.g., “looking forward to editing my photographs and naming the wildlife I have seen” [P107] or about recounting the day to others.

Our questions asked people to recall short-term wetland visits so happiness/pleasure (as an expression of subjective wellbeing [141]) might only be approached in terms of affective, hedonic or experienced wellbeing [142]. Although eudemonic wellbeing traits, such as seeking to elevate experiences (e.g., awe, inspiration and sense of connection with a greater whole) among respondents may also have been a motivation [143]. This widespread expression of subjective wellbeing reflects other UK research that found that participants using a phone application to map subjective wellbeing to spatial data were substantially happier outdoors in all green or natural habitat types compared with urban environments [144]. Studies of positive experiential wellbeing have also shown that specific and recent (i.e., yesterday) nature visits are associated with greater happiness [145].

Negative feelings (7 MUs, 0.7%) were in the minority and related to frustrations at missed wildlife sightings. Successfully executed wildlife sightings have parallels to peak experience [146], flow states [147] and consequent benefits to wellbeing—but only when the challenge is met by individual competence [54]. Where there is a deficit in competence, frustration can inhibit fulfilment and the inexperienced can become anxious at missed sightings due to perceived shortcomings [54]. While our results present an overall positive wellbeing picture for wildlife watching, these examples of frustrations highlight some of the potential non-benefits to wellbeing regarding wildlife experiences.

#### 3.2.2. Physical Derived Effects

The physical domain, relating to the physical body, was the second-most referenced domain (248 MUs, 23.7%). The theme of relaxation was the dominant theme, and to feel relaxed was the single most frequent response across all during-visit derived effects (176 MUs, 16.8%). This supports the finding that relaxation is a central benefit of freshwater blue spaces [61]. Post-visits, feeling relaxed was the most endorsed code to that question (103 MUs, 12.2%; Table 3). The related code of reduced stress also emerged in equal measures both during (13 MUs, 1.2%) and post-visits (12 MUs, 1.4%). This capacity of natural environments to reduce stress, to relax and to provide psychological restoration is well theorised [1,70]. Research on urban situated wetland centres (of the type surveyed for the present study), in comparison with urban environments, has demonstrated the potential of wetland centres to mitigate stress and to improve mood with just ten minutes of nature exposure [73]. In that study, the effects were shown to be more pronounced for people self-reporting elevated stress, complementing other work on urban green spaces that demonstrated that stressed individuals are motivated to use green spaces more often [148]. Where derived effects have been modelled and linked to motivations to visit natural environments, the motivation to reduce stress most strongly explains restorative experiences and positive post-visit mental states (compared with the other motivations: physical activity, solitude, social interactions and experiences with nature) [139]. Many of the participants were motivated by the nature and birdlife offered, and these relaxation/stress recovery effects support other research that demonstrates that the relaxation benefits of freshwater blue spaces is especially important for people to whom nature is important [61] and that bird interactions and greater species richness is associated with perceived stress recovery, psychological wellbeing and attention restoration [5,149,150,151].

The theme of revitalisation (48 MUs, 4.6%; Table 3) focuses on replenishing energy and is distinct from relaxation. This was expressed in broader terms, and these were organised as refreshed (including renewed, recuperated, and revived), full of fresh air (including “being able to breath” [P65]), energised and exercised. There was a notable doubling of feelings relating to revitalisation in post-visit responses (97 MUs, 11.5%; Table 3). There is an important distinction between revitalisation and relaxation. Here, we group relaxation and revitalisation within the physical domain (following Irvine et al. [43]) but note that other researchers assess vitality—defined as ‘a positive activation state of having energy available for oneself, both psychologically and physically’—as an affective outcome of nature visits [139,152]. There is some evidence that natural environments can lead to additional wellbeing benefits through improved vitality [118,153]. Part of that response is likely to feature contributions from social interactions and physical activity, but other characteristics of outdoor environments, such as the intangible aspects of nature (e.g., fresh air or open space), also play a role [153]. As noted, physical activity is less of a motivator for wetland centres, whereas intangible nature is more prominent; thus, tentatively, we suggest that the latter may also play a role in delivering revitalising effects. The increase in feelings of revitalisation post-visit is also suggestive of a lag associated with revitalisation benefits and raises questions about how long health benefits of nature exposure might last [21,154,155].

#### 3.2.3. Cognitive Derived Effects and Attention Restoration Theory

The cognitive domain was the third-most mentioned (179 MUs, 16.9%) and is underpinned by attention restoration theory (ART) [71]. Within this domain, the theme of ART was dominant (132 MUs, 12.6%), with evidence from across the sample of how wetland centres reflect all four theorised qualities of restorative environments, i.e., being away (escape from everyday concerns), extent (being in a whole other world) of compatibility (finding activities that are ‘compatible’ with intrinsic motivations) and soft fascination (involuntary or effortless attention) [71,72,139].

Being away (39 MUs, 3.7%) was expressed diversely in terms of psychological and physical escape, e.g., “problems fall away when surrounded by, and totally involved with, nature” [P331] and “getting away from all the trappings of modern civilisation” [P243]. Within this code, elements of extent emerged through the expression of escape to other worlds, e.g., “time stands still and it is like the rest of the world is far away” [P198], “away from all life’s problems transported to another world” [P324] and “miles away from everyday life lost in her world.” [P325]. Further appreciation of the refuge provided from the city environment was also expressed through the ‘contrast to city’ place attachment code. Escape from day-to-day life and concepts of extent are important explanatory factors in the success of wetland nature-based health interventions, with participants relating being away from day-to-day life to reductions in the symptoms associated with their anxiety and/or depression [20]. Other nature-based health interventions and volunteering reported similar benefits [36,156]. Escape from everyday life and work is an important aspect to users of Finnish protected areas [56]. While other studies have recorded the importance of urban forests in allowing users to be away ‘to’ nature as well as away ‘from’ everyday stressors [115]. These findings align with other wetland specific research that finds concepts of attention restoration important for the quality of life of people living near urban wetlands [78]. By contrast, few respondents (17 out of 527 statements (3.2%)) directly mentioned mental restoration in similar research on UK park spaces [43].

The capacity of the wetland centres to create interest/fascination was captured within the theme of attention restoration as respondents referred to being interested (28 MUs, 2.7%; fascinated, enthralled or curious), focused (20 MUs, 1.9%; concentration, in the moment, absorbed and alert), engaged (15 MUs, 1.4%), inspired (7 MUs, 0.7%) and motivated (6 MUs, 0.6%). There remains a question regarding how much of this fascination is ‘soft’ (involuntary) or ‘hard’ (voluntary). We have been unable to link motivations to derived effects although the strength of the nature-related motivations implies that, for many, this interest is linked to motivations regarding experiences with nature. In a study that did link motivation, attention focus and effects, the authors tentatively suggested that, for visitors motivated to experience nature, a directed attentional focus on the environment is needed for positive wellbeing outcomes [139]. Fascination—and the distraction offered by wetland biota—has been cited as a possible route for the observed positive health outcomes observed in wetland nature-based health interventions [20]. Most responses regarding fascination within our data were stated singly and without context, restricting insights on the wider context to where interest, focus and/or engagement was directed.

Additionally, within the cognitive domain was the theme of satisfaction (35 MUs, 3.3.%), often singularly expressed and largely without much context, e.g., “content” [P96], “satisfied”, [P256], “fulfilled” [P329] and “rewarded” [P365]; those that were more descriptive mentioned contentment of the visitor and wildlife experience, e.g., “I have yet to feel disappointment on any visit” [P18] or wildlife, e.g., “content being amongst the wildlife” [P308]. Post-visit, the theme of feeling satisfied emerged to a greater extent than during visits (82 MUs, 9.7%), with similar post-visit endorsements for feeling relaxed (91 MUs, 11.1%) and happy (86 MUs, 10.1%). This post-visit contentment aligns with ART’s compatibility requirement and the need to find activities and experiences that are compatible with intrinsic motivations [71,72].

#### 3.2.4. Spiritual Derived Effects

The spiritual domain (157 MUs, 15.0%) is similar in tone to the theme of attention restoration. It captures the feelings of calm (60 MUs, 5.7%), peacefulness (44 MUs, 4.2%) and being at ease (11 MUs, 1.0%); the latter included mention of being “at peace with myself” [P224] or at “ease with the world” [P192]. Feeling connected to nature (29 MUs, 2.8%) exemplified being “at one with” [P37], “in touch with” [P101], “engaged with” [P356] or “closer to” [P76] nature. Others were more descriptive of their spirituality, e.g., “it’s a chance to let my senses go and listen, smell, and see the wonders of nature” [P337], “a feeling that this is a place where I feel connected both to non-human animals and plants” [P336] and “it helps me to realise my belonging with other creatures” [P193]. Others provided more practical and proximal descriptions, e.g., “feeding the ducks, listening to the Eiders” [P295] or “pure enjoyment at the proximity of birds and nature” [P302]. Others wrote of familiarities “I know some of their [swans] story and it is important to me that they are there” [P134]. These descriptions resonate with the findings that interacting with birds is associated with improved general wellbeing, relaxation, connection to nature and pro-environmental behaviours [157,158], and perceived stress recovery and attention restoration [159]. The spiritual domain was less prominent post-visit (62 MUs, 7.1%) due to reductions in the expressions of tranquillity and connection to nature as respondents recalled the reduced intimacy of nature post-visits.

Spirituality is an important and underrepresented aspect of the ecosystem service agenda [160,161] and receives less research attention in comparison with the physical, cognitive and psychological aspects [162]. It has however been positively related to and been found to mediate exposure to nature and wellbeing [163]. Our results somewhat dilute the importance of spiritually through the coding process, and this may well be more important than is presented within the spirituality theme. For example, responses such feeling uplifted, inspired (grouped as a cognitive benefit under ART) and amazed (grouped as affective) were also recorded across the taxonomy. These chime with the “awe, wonder and privilege” elicited by nature encounters in wildlife tourists through ethnography [54] or the awe and calming effects reported for wild cliffs and manicured gardens [164]. These findings have relevance for the conservation sector not only through wellbeing but also through the increased connection to nature, which is increasingly proposed as a method to garner public support for environmental protections [165,166]. As Curtin [54] observed, the spiritual aspects of wildlife experiences not only inspire but also can induce a deep sense of wellbeing and psychological health benefits.

#### 3.2.5. Place Attachment Derived Effects

The place attachment domain (114 MU, 10.9%) collated respondents’ emotions towards the wetland centre sites. The theme of site value (59 MUs, 5.6%) grouped broad-scale positive responses regarding a pleasant/enjoyable space. Feelings of safety, both emotional and physical, as well as recognition of a valued contrast to the city/urban environment were also recorded. Appreciation (29 MUs, 2.8%) of the sites was expressed mainly in terms of gratitude for the conservation of the site and wildlife. Negative comments that detracted from the experience (26 MUs, 2.5%) focused on frustrations with site management, the behaviour of other visitors (mainly children) and crowding. The endorsement of enjoyment and appreciation for the site and the experience mirror responses recorded as derived effects by users of UK urban park spaces [43]. While comparisons between New York’s ‘natural’ and landscaped park areas showed that place attachment themes can vary depending on environment, in this example, they were more prevalent in natural areas than the landscaped ones [48].

After visits, the place attachment domain had a much greater salience and was the second-most endorsed domain (172 MUs and 20.4%). A theme of interactional place attachment emerged that was not observed for during visits [167]. This theme captures a) the anticipation of future visits and b) an appreciation of the memories created, induced through respondents looking forward to/planning their next visit (38 MUs, 4.4%) and thinking about what they will do/see the next time, e.g., “I am often thinking about what I will do next time” [P166]. The interactional past was expressed through references to memories (11 MUs, 1.3%) formed by—and the pleasant recall of—the experiences, e.g., “(I) treasure the memories when I am back at work” [P97].

This grouping was based on an interactional theory of place attachment (from the built environment) that describes the interactional past (past experiences with a site, or ‘memories’) and the interactional potential of a site (‘future experiences imagined or anticipated’) [167]. Future potential is an important facet to the nature and wellbeing agenda, and it can extend and sustain the wellbeing impacts of nature engagement beyond the immediate experience. Memories are an important but understudied facet of the psychological benefits of place attachment [168]. Memories of our favourite places have health benefits through enhanced place identity and greater perceptions of the restorative capacity of those places [169]. Furthermore, natural favourite places have been shown to remain stable—as a favourite place—over time [170], and this stability is an important feature for psychological self-regulation and for the maintenance of self-esteem [171]. For meaningful wildlife experiences, memories and reflection on a memory can add value to lives long after the visits [172] and can create deeper emotional connections to the experiences had [173]. As Curtin [54] notes, “wildlife experiences whether on tour or at home can sustain the human spirit. The knowledge that it is all there waiting to be discovered and the memory of past sightings builds hope and expectations of the next occurrence”. Evoking memories maybe harnessed as a management option for site managers looking to enhance environmentally responsible behaviours—the options were explored in more detail by [174] but some examples may include enhancing multi-sensory experiences, facilitating close encounters with wildlife, or using interpretive commentaries and signage. Stimulating sensory impressions is also a prerequisite for the health-promoting properties of local green environments in Sweden [175]. Evoking nature-based memories might find application as a treatment for mental health conditions including depression and anxiety [176].

Research on the relationship between people, places and nature is beginning to reveal the importance of place attachment to human wellbeing and the mediating role played by nature connection [177,178,179]. Place attachment may contribute to as a much as 30% of the total effect of nature connectedness on wellbeing (taken from a study of n = 2203 Japanese nationals) [179]. Although the results are mixed, there is some evidence for moderate positive effects of place attachment to pro-environmental behaviours [180]. Taken together, place attachment represents an interesting construct to explore for nature managers looking to improve wellbeing, connection to nature and pro-environmental behaviours [181].

#### 3.2.6. Social Derived Effects

The social domain (45 MUs, 4.3%) emerged through the mention of feeling connections to others (25 MUs, 2.4%), mainly through an appreciation for the opportunity for time spent with friends and family, feelings of familiarity and feeling at home, e.g., “it’s like coming home” [P346]. This effect had relatively low salience, and for UK park spaces, this effect was even less well endorsed [43]. This sits in contrast to panel surveys conducted in the UK that found that 33% of people reported that spending time with friends was the single most important benefit they received by visiting freshwater blue spaces [61]. This study also found that socioeconomic differences were predictors for identifying social interaction as the most important benefit received from visiting blue spaces, serving as a reminder that the use and benefits derived of blue/green spaces may vary for different socioeconomic groups.

#### 3.2.7. Global Wellbeing Derived Effects

The final domain (13 MUs, 1.2%) of global wellbeing included deliberate mention of feeling better, and health or wellbeing improvements, e.g., “[I] feel better” [P109], “improved mood” [P141], “fit” [P211], “healthy” [P150], “a sense of wellbeing” [P131] and more descriptively “overall simply doing something which is good for me” [P278].

### 3.3. Limitations

The limitations to this work relate to the sample population, data collection and coding process. We took advantage of existing visitor communication networks to generate good engagement with the survey (50% response rate). Although our sample is representative of typical wetland centre visitation, the supporter base is not representative of the wider UK population. By sampling existing visitors and supporters, we can also expect a degree of existing connections to nature and pro-nature bias in the responses regarding visitor experiences. Furthermore, a high proportion of respondents were members of the nature organisation, and bias may have been introduced through respondents’ display of demand characteristics and a desire to support the organisation and nature conservation [182], notwithstanding the presence of negative comments within the data. Use of, and the derived benefits from, natural environments are known to vary across socioeconomic, gender and health inequalities gradients [61,183]. Here, we drew comparisons to UK park spaces [43]; however our extrapolations are limited by differences in the sample populations inherent to the environment offer, i.e., paid-for visitor attractions versus free-to-use public spaces.

The online self-administered survey has an associated inability to consider any latent, non-verbal data. Therefore, the findings are based on manifest data alone (i.e., that which is contained within the text) [97]. We also asked people to recall past feelings, and these responses are likely to include some overestimation of a past affect [184]. Our inter-reliability testing found good consistency; however, the responses given may have been interpreted differently by the researchers, with differing personal histories likely to influence interpretation in the coding process [97]. Finally, our coding framework was based on and developed from similar work that surveyed UK urban green space users [43]. Although this grounded the process, provided time efficiencies and enabled us to make comparisons between UK urban park space and wetland centre environments, the limitations from that framework should also be considered [43].

## 4. Conclusions

With the combined global health challenges around mental health disorders, NCDs and COVID-19 [13,14,15,23,24,25], demand is growing to understand the degree to which experiences with nature can be harnessed for public health benefit and how reciprocal benefits for biodiversity might be delivered through a health agenda [42,83]. As such, there is a need for evidence on the impacts of a broad range of natural environments on the health of users of these environments [94,95].

Our results show the diversity and prominence of motivations and derived effects through weighted taxonomy associated with active users of wetland centres. Motivation to visit is strongly influenced by the quality of the space, most prominently the biological nature (i.e., the wildlife). Abiotic and intangible aspects of nature as well as good amenities were also important factors. In contrast to open-access, green spaces, physical activity was a lower-level motivator for use [1,43,118], representing one area of focus for future design/management of wetland centres looking to improve their impact as a health resource. We also found that mental restoration was a prominent motivation to visit and effect, especially with respect to being away, suggesting that concepts of attention restoration theory may be important to the wetland visitor experience [71,72]. For site management, these cognitive benefits may offer a fertile ground for both conservation and public health visitor marketing [138].

Although mainly motivated by the quality of the space, for our sample set (i.e., positively nature-orientated, freshwater blue space visitors), wetland centres are places that provide psychological wellbeing by inducing feelings of happiness/pleasure, relaxation, mental restoration, vitality and satisfaction. This echoes much of the body of literature on natural environments and mental wellbeing [4,5,6,185] and provides further evidence that these environments could be suitable for nature-based health interventions or nature prescriptions for combating mental health problems [16,20,36,186]. In response to the global health challenges outlined above, this idea remains a promising and advanced mechanism for harnessing nature for health [187,188].

Our findings must be considered in the context that different socioeconomic groups experience and derive benefits from nature differently [61,64,189]. However, these results imply that, for decision-makers looking to leverage blue space for public health benefit, for the nature-orientated at least, improving the quality of the space may be one route towards health gains while also bringing co-benefits for the environment and biodiversity [38,83].

## Figures and Tables

**Table 1:** Recalled participant motivations to visit UK wetland visitor centres from *n* = 365 participant responses to the question “What are your main reasons for visiting a WWT Wetland Centre?” via an online survey. The number of assigned meaning units (MUs) per domain, theme or code are presented along with the percentage of total MUs for the question (*n* = 1522).

Domain	No.	Theme	No.	Code	No.	Sub-Code		No.
Space qualities	946 (62.2%)	Nature	568 (37.3%)	Birds	237 (15.6%)	Bird watching		188 (12.4%)
						Specific bird group		14 (0.9%)
						Interesting birds		12 (0.8%)
						Not captive		12 (0.8%)
						Identify birds		9 (0.6%)
						Rare bird		2 (0.1%)
				Wildlife	129 (8.5%)			
				Get outside	58 (3.8%)			
				Fresh air	45 (3.0%)			
				Seasons	35 (2.3%)			
				Pleasant environment	24 (1.6%)			
				Wetlands/water	12 (0.8%)			
				View (scenery)	8 (0.5%)			
				Protected nature	9 (0.6%)			
				Flora	7 (0.5%)			
				Insects	4 (0.3%)			
		Site features	353 (23.2%)	Site amenities	127 (8.3%)	Café		37 (2.4%)
						Living collection		33 (2.2%)
						Hides		27 (1.8%)
						Shop		19 (1.2%)
						Facilities		11 (0.7%)
				Site management	99 (6.5%)	Day out		25 (1.6%)
						Activities		23 (1.5%)
						Visitor experience		16 (1.1%)
						Site design		11 (0.7%)
						Staff		24 (1.6%)
				Peace and quiet	52 (3.4%)			
				Proximity	17 (1.1%)			
				Close wildlife	17 (1.1%)			
				Accessible	12 (0.8%)			
				Safe	9 (0.6%)			
				Atmosphere	6 (0.4%)			
				Nuisance-free	7 (0.5%)			
				Open space	7 (0.5%)			
		Place attachment	17 (1.1%)	Emotional attachment	17 (1.1%)			
		Place identity	8 (0.5%)	History of use	8 (0.5%)			
Cognitive	171 (11.2%)	Mental pursuits	100 (6.6%)	Photography	44 (2.9%)			
				Learn	33 (2.2%)			
				Purposeful work	23 (1.5%)			
		Attention restoration	71 (4.7%)	Being away	46 (3.0%)			
				Mental health	25 (1.6%)			
Physical	161 (10.6%)	Physical restoration	97 (6.4%)	Relax	58 (3.8%)			
				General wellbeing	16 (1.1%)			
				De-stress/unwind	15 (1.0%)			
				Eat/drink	8 (0.5%)			
		Physical pursuits	64 (4.2%)	Walking	44 (2.9%)			
				Exercise	20 (1.3%)			
Social	71 (4.4%)	Existing	53 (3.5%)	Time with friends/family	39 (2.6%)			
				Friendly people	14 (0.9%)			
		New	14 (0.9%)	Meet like minds	14 (0.9%)			
Children	67 (4.5%)	Nature	18 (1.2%)	Nature experience	18 (1.2%)			
		Social	18 (1.2%)	With adult	18 (1.2%)			
		Physical	11 (0.7%)	Play	11 (0.7%)			
		Child Development	21 (1.4%)	Wildlife interest	10 (0.7%)			
				Learn	11 (0.7%)			
Spiritual	53 (3.5%)	Interconnected	53 (3.5%)	Connected to nature	53 (3.5%)			
Values	56 (3.7%)	Altruism	56 (3.7%)	Support conservation	56 (3.7%)			
Total Meaning Units	1522 (100%)							
Removed	10							

**Table 2:** Recalled participant-derived effects to visit UK wetland visitor centres from *n* = 365 participant responses to the question “Thinking about when you are at a WWT Wetland Centre, please describe how you feel during your visit” via an online survey. The number of assigned meaning units (MUs) per domain, theme or code are presented along with the percentage of total MUs for the question (*n* = 1048).

Domain	No.	Theme	No.	Code	No.
Affective	292 (27.9%)	Positive emotions	192 (18.3%)	Happy	98 (9.4%)
				Pleasant	42 (4.0%)
				Anticipation	27 (2.6%)
				Hopeful	8 (0.8%)
				Privileged	7 (0.7%)
				Positive	5 (0.5%)
				Good/fine/nice	5 (0.5%)
		Intensely positive emotions	93 (8.9%)	Excited	65 (6.2%)
				Great pleasure	17 (1.6%)
				Amazed	11 (1.0%)
		Negative emotions	7 (0.7%)	Frustration(at missed sighting)	7 (0.7%)
Physical	248 (23.7%)	Relaxed	176 (16.8%)	Relaxed	163 (15.6%)
				Reduced stress	13 (1.2%)
		Revitalised	48 (4.6%)	Refreshed	18 (1.7%)
				Full of fresh air	14 (1.3%)
				Energised	11 (1.0%)
				Exercised	5 (0.5%)
		Comfort	18 (1.7%)	Comforted	8 (0.8%)
				Uncomfortable	6 (0.6%)
				Rested	4 (0.4%)
		Weathered	6 (0.6%)	Weathered	6 (0.6%)
Cognitive	179 (17.1%)	Attention restoration	132 (12.6%)	Being away	39 (3.7%)
				Interested	28 (2.7%)
				Focused	20 (1.9%)
				Engaged	15 (1.4%)
				Better perspective	12 (1.1%)
				Inspired	7 (0.7%)
				Motivated	6 (0.6%)
				Clear headed	5 (0.5%)
		Satisfied	35 (3.3%)	Satisfied/content	35 (3.3%)
		Educated	12 (1.1%)	Educated	12 (1.1%)
Spiritual	157 (15.0%)	Tranquillity	115 (11.0%)	Calm	60 (5.7%)
				Peaceful	44 (4.2%)
				At ease	11 (1.0%)
		Interconnected	29 (2.8%)	Connected to nature	29 (2.8%)
		Improved spirit	13 (1.2%)	Uplifted	13 (1.2%)
Place attachment	114 (10.9%)	Value of site	59 (5.6%)	Nice experience	18 (1.7%)
				Nice space	11 (1.0%)
				Enjoyable Place	12 (1.1%)
				Safe	9 (0.9%)
				Contrast to city	9 (0.9%)
		Appreciation	29 (2.8%)	Pleased	8 (0.8%)
				Grateful	11 (1.0%)
				Appreciation	10 (1.0%)
		Detractors from the site experience	26 (2.5%)	Site management	9 (0.9%)
				Behaviour	9 (0.9%)
				Crowds	8 (0.8%)
Social	45 (4.3%)	Connected to others	25 (2.4%)		
		Familiar	11 (1.0%)		
		Welcome	9 (0.9%)		
Global wellbeing	13 (1.2%)	Better	8 (0.8%)		
		Healthy	5 (0.5%)		
Total Meaning Units	1048 (100%)				
Removed	13				

**Table 3:** Recalled participant-derived effects to visit UK wetland visitor centres from n = 365 participant responses to the question “Thinking about when you leave a WWT Wetland Centre, please describe how you feel after your visit” via an online survey. The number of assigned meaning units (MUs) per domain, theme or code are presented along with the percentage of total MUs for the question (*n* = 843).

Domain	No.	Theme	No.	Code	No.
Physical	251 (29.8%)	Relaxed	103 (12.2%)	Relaxed	91 (10.8%)
				Reduced stress	12 (1.4%)
		Revitalised	97 (11.5%)	Refreshed	52 (6.2%)
				Energised	28 (3.3%)
				Full of fresh air	11 (1.3%)
				Exercised	6 (0.7%)
		Weathered	7 (0.8%)	Weathered	7 (0.8%)
		Depleted	44 (5.2%)	Tired	32 (3.8%)
				Rested/fed	4 (0.5%)
				Apprehension for drive ahead	8 (0.9%)
Place attachment	172 (20.4%)	Value of site	72 (8.5%)	Sad because leaving	35 (4.2%)
				Nice experience	28 (3.3%
				Enjoyable place	9 (1.1%)
		Interactional	47 (5.6%)	Looking forward to next visit	36 (4.3%)
				Memories	11 (1.3%)
		Appreciation	46 (5.5%)	Pleased	24 (2.8%)
				Glad	14 (1.7%)
				Grateful	8 (0.9%)
		Detractors from the site experience	7 (0.8%)	Site management	5 (0.6%)
				Crowds	2 (0.2%)
Affective	162 (19.2%)	Positive emotions	126 (14.9%)	Happy	86 (10.2%)
				Anticipation	15 (1.8%)
				Pleasant	12 (1.4%)
				Good/fine/nice	9 (1.1%)
				Hopeful	4 (0.5%)
		Intensely positive emotions	25 (3.0%)	Excited	16 (1.9%)
				Great pleasure	9 (1.1%)
		Negative emotions	9 (1.1%)	Frustration (site access)	5 (0.6%)
				Frustration (at missing sighting)	4 (0.5%)
		Neutral emotions	2 (0.2%)	Do not know how I feel	2 (0.2%)
Cognitive	158 (18.7%)	Satisfied	91 (10.8%)	Satisfied/content	82 (9.7%)
				Sense of accomplishment	9 (1.1%)
		Attention restoration	67 (7.9%)	Motivated	23 (2.7%)
				Educated	10 (1.2%)
				Being away	9 (1.1%)
				Interested	8 (0.9%)
				Inspired	5 (0.6%)
				Enriched	4 (0.5%)
				Better perspective	8 (0.9%)
Spiritual	59 (7.0%)	Tranquillity	35 (4.2%)	Calm	25 (3.0%)
				Peaceful	10 (1.2%)
		Connectedness with nature	11 (1.3%)	Connected to nature	11 (1.3%)
		Improved spirit	13 (1.5%)	Uplifted	13 (1.5%)
Global wellbeing	21 (2.5%)	Better	17 (2.0%)		
		Healthy	4 (0.5%)		
Social	16 (1.9%)	Connected to others	16 (1.9%)		
Values	4 (0.5%)	Support conservation	4 (0.5%)		
Total Meaning Units	843 (100%)				
Removed	9				

## Data Availability

To protect participant anonymity ethical approval (WWT0325112019) prevents data sharing.

## Supplementary Materials

The following are available online at https://www.mdpi.com/article/10.3390/ijerph18168629/s1, Table S1. Demographics and organisation affiliation of the survey respondents, Table S2. Example conversions of the participants’ full text to short phrase meaning units (MUs), Table S3. Inter-rater reliability scores for each question using Cohen’s kappa value (McHugh 2012).
