# Supplementary material for: A Qualitative Analysis of UK Wetland Visitor Centres as a Health Resource
Source: Int J Environ Res Public Health. 2021 Aug 15;18(16):8629. doi: 10.3390/ijerph18168629 (PMC8392124; doi:10.3390/ijerph18168629)
Supplement: Supplementary file 1 [file ijerph-18-08629-s001.zip › ijerph-1302481-supplementary.pdf]

International Journal of
Environmental Research and Public Health

MDPI

Table S1 Demographics and organisation affiliation of survey respondents

Factor		Frequency	% of Participants
Gender	Male	190	49.4
	Female	195	50.6
Age	20-29	1	0.3
	30-39	1	0.3
	40-49	20	5.2
	50-59	40	10.4
	60-69	84	21.8
	70-79	136	35.3
	80-89	84	21.8
	Prefer not to say	17	4.4
Ethnicity	Mixed / Multiple ethnic groups	2	0.5
	Other ethnic group	2	0.5
	Prefer not to say	7	1.8
	White / White British / White Irish	374	97.1
Membership	Prefer not to say	1	0.3
	I am a WWT member	349	90.6
	I am no longer a WWT member but have been in the past	14	3.6
	I have never been a WWT member	21	5.5
Frequency of Visits in the last year	0	15	3.9
	1-5	182	47.3
	6-15	141	36.6
	16 - 25	23	6.0
	26+	21	5.5

Table S2 Examples conversion of participant full text to short phrase meaning units (MUs)

Original Response	Meaning Unit
Bird watching and some fresh air! [P199 – Motivations question]	Bird watching<br>Fresh air
We find it calming most of the time, but spotting the kingfisher always makes the pulse race a little. It’s lovely to see the pleasure seeing this delightful bird brings to others. [P191 – During effects question]	Calming most of the time<br>But spotting the kingfisher always makes the pulse rate a little<br>It’s lovely to see the pleasure seeing this delightful bird brings to others
I'd say I'm mostly happy and relaxed and glad for a nice time having been had. [P157 – During effects question]	Mostly happy<br>Relaxed<br>Glad for a nice time

Table S3 Inter-rater reliability scores for each question, using Cohen's Kappa Value (McHugh 2012)

Question	Percentage of MUs Jointly Coded	Agreement Scores (%)	Cohen's Kappa Value
What are your main reasons for visiting a WWT Wetland Centre?	21.2	71.62	0.706
Thinking about when you are at a WWT Wetland Centre, please describe how you feel *during* your visit…	20.7	86.73	0.860
Thinking about when you leave a WWT Wetland Centre, please describe how you feel *after* your visit…	20.3	87.30	0.861
